# Supplementary material for: Interferon-λ treatment accelerates SARS-CoV-2 clearance despite age-related delays in the induction of T cell immunity
Source: Nat Commun. 2022 Nov 16;13:6992. doi: 10.1038/s41467-022-34709-4 (PMC9667439; doi:10.1038/s41467-022-34709-4)
Supplement: Supplementary file 1 — Supplementary Information [file 41467_2022_34709_MOESM1_ESM.pdf]

Supplementary Table 1. scRNA sequencing patient characteristics

	Placebo	PEG-IFN-λ	Total
**Total #**	4	5	9
**Sex**			
Female	2	3	5
Male	2	2	4
**Median age, years (range)**	47 (25-63)	47 (27-60)	47 (25-63)
***IFNL4* genotype**			
ΔG	0	0	0
TT/ΔG	1	2	3
TT	3	3	6

Supplementary Table 2. List of ISGs used in ISG score computation

**ISG**
IFIT1
IFI6
ISG15
OAS1
MX1
OASL
STAT1
JAK1
IFI27
IFITM1
IFITM2
IFITM3
IFI44L
B2M
CD83
DDIT4
EHD4
IFIT2
IRF1
NFKBIZ
NXPE3
PIM3
RSG2
TNFSF10

Supplementary Table 3. RBD-specific Ig correlations. P-values based on two-sided Spearman rank correlation tests with no adjustments for multiple comparisons.

Correlations (Spearman r)

**D7**	IgG	IgA	IgM	**D90+**	IgG	IgA	IgM
IgG				IgG			
IgA	0.424*			IgA	0.323		
IgM	0.790****	0.624***		IgM	0.108	0.124	

* = correlation p < 0.05
*** = correlation p < 0.001
**** = correlation p < 0.0001

Supplementary Table 4. RBD-specific IgG correlations with spike T cell responses. P-values based on two-sided Spearman rank correlation tests with no adjustments for multiple comparisons.

Correlations (Spearman r)

**D0**	IgG	IgA	IgM	**D7**	IgG	IgA	IgM	**D90+**	IgG	IgA	IgM
IFN-γ	0.30	0.388	0.196	IFN-γ	0.277	0.112	0.165	IFN-γ	**0.455***	**0.547****	0.142
IL-2	0.336	0.207	0.057	IL-2	0.128	0.027	0.015	IL-2	**0.556****	**0.495***	0.004
Poly	0.178	0.327	0.160	Poly	0.262	0.094	0.156	Poly	**0.512***	**0.554****	0.056

* = correlation p < 0.05
** = correlation p < 0.01

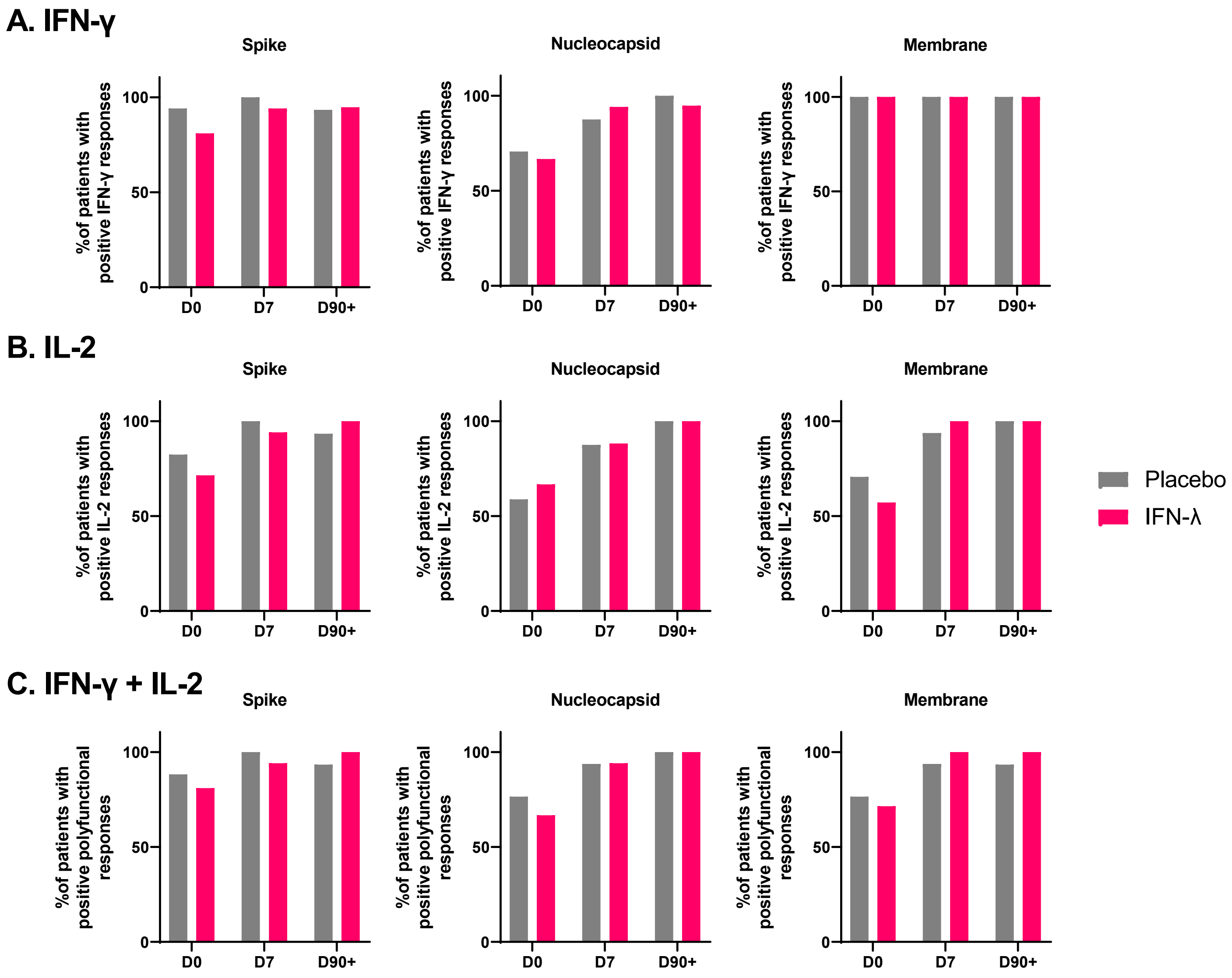

Supplementary Figure 1. Percentage of positive T cell responses towards SARS-CoV-2 structural proteins across D0, D7, and D90+. A) IFN-γ B) IL-2 and C) polyfunctional (IFN-γ + IL-2) T cell responses towards Spike, Nucleocapsid, and Membrane were determined by FluoroSpot assay, with positive responses defined as the number of spot forming units (SFUs)/million exceeding twice the individual's negative control SFU count and greater than the mean negative SFU count from all patients. N values are as follows: Placebo, D0 (n = 17), Placebo, D7 (n = 16), Placebo, D90+ (n = 15), IFN-λ, D0 (n = 21), IFN-λ, D7 (n = 17), IFN-λ, D90+ (n = 19). No significant difference in proportions between treatment groups was observed using a two-sided Chi-square test with Yates' correction. Source data are provided as a Source Data file.

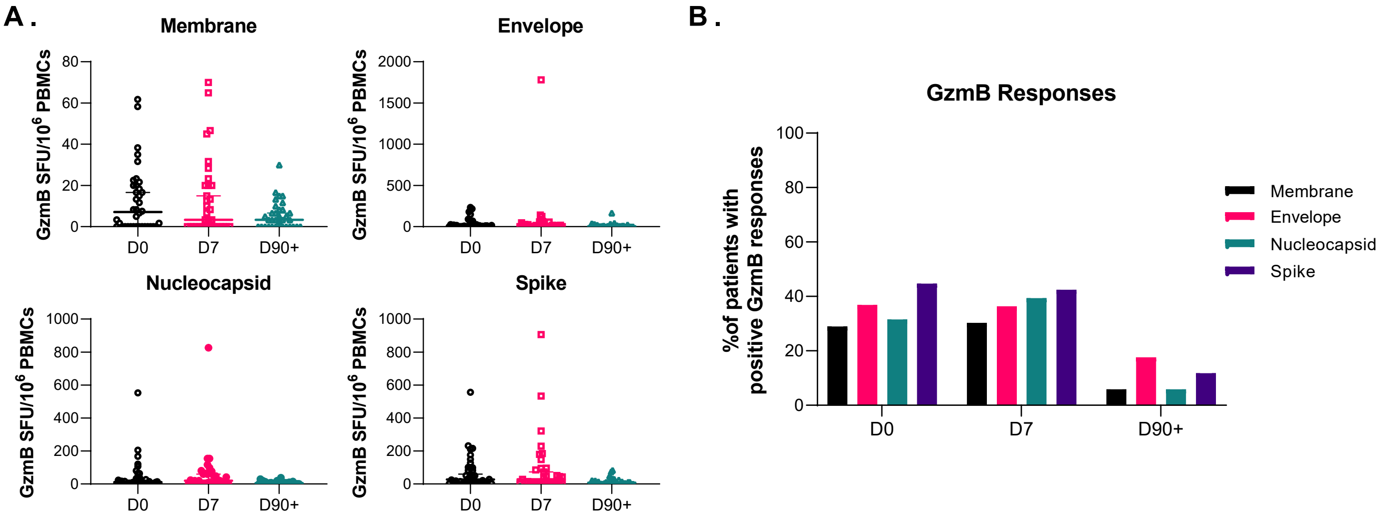

Supplementary Figure 2. Granzyme B T cell responses to SARS-CoV-2 structural proteins. A) The number of SFUs/10^6 PBMCs according to each SARS-CoV-2 protein B) The percentage of patients with a positive granzyme B (GzmB) response towards the SARS-CoV-2 proteins. Each dot represents a different patient. N values are as follows: D0 (n = 38), D7 (n = 33), D90+ (n = 34). No significant differences between groups were observed using two-sided Mann-Whitney U tests. Bar lines represent median and 95% CI. Source data are provided as a Source Data file.

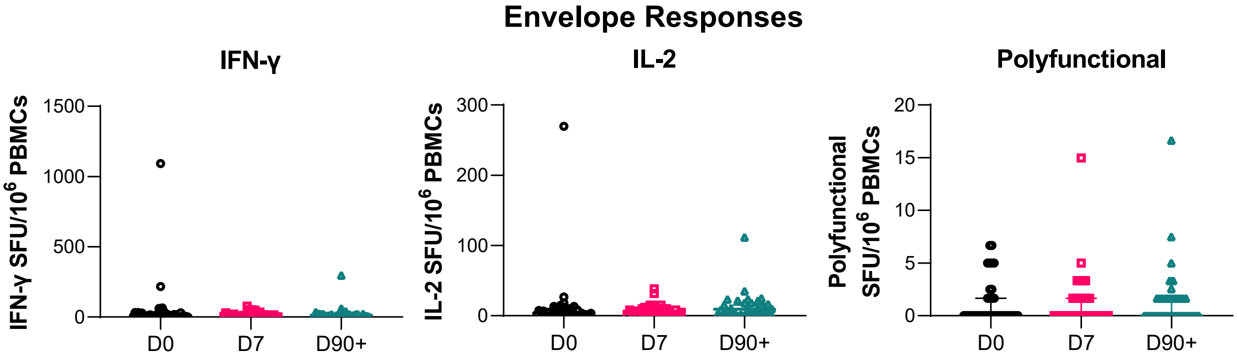

Supplementary Figure 3. T cell responses to SARS-CoV-2 envelope protein. IFN-γ, IL-2 and polyfunctional (IFN-γ + IL-2) T cell responses (as SFUs per 10^6 PBMCs) against SARS-CoV-2 envelope peptide pools were quantified *ex vivo* using FluoroSpot assays. Each dot represents a different patient. N values are as follows: D0 (n = 38), D7 (n = 33), D90+ (n = 34). No significant differences between groups were observed using two-sided Mann-Whitney U tests. Bar lines represent median and 95% CI. Source data are provided as a Source Data file.

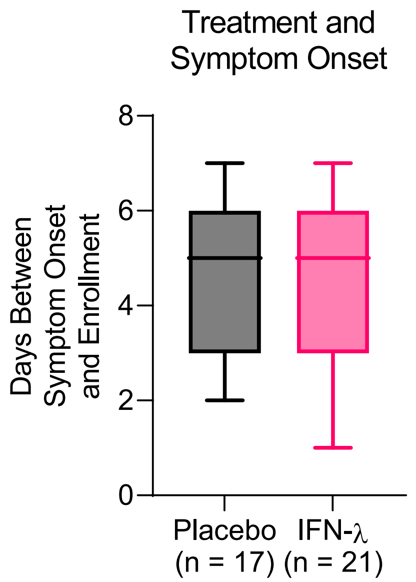

Supplementary Figure 4. Differences in the time between symptom onset and enrollment in different treatment groups. Only significant differences between groups are shown (Two-sided Mann-Whitney U-test). Tukey boxplots are shown, where outer bounds of the box represent the first and third quartile, the centre line represents the median, and whiskers represents the nearest rounded whole number from 1.5 times the IQR below the first quartile and above the third quartile. Minima and maxima correspond with the whiskers in this instance. Source data are provided as a Source Data file.

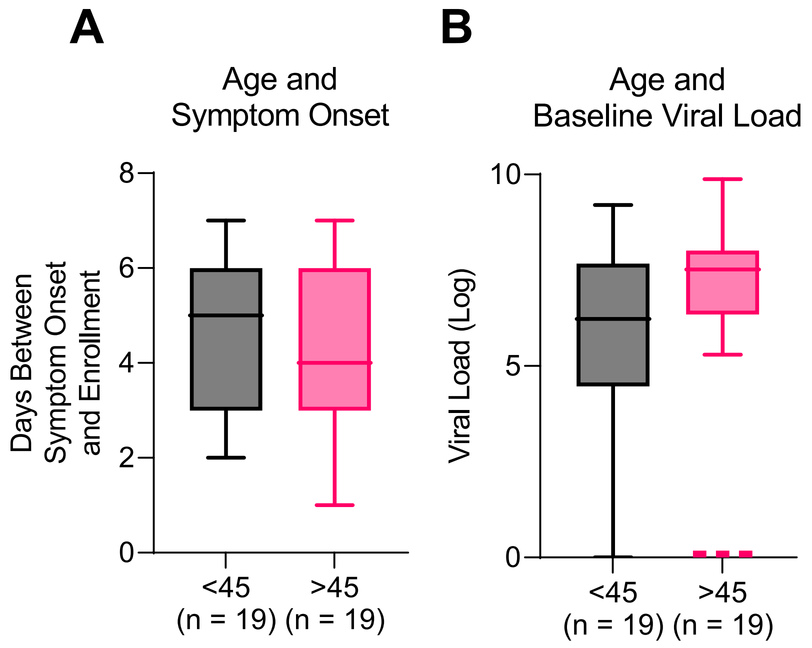

Supplementary Figure 5. Differences in age and A) the time between symptom onset and enrollment and B) baseline viral load. Only significant differences between groups are shown (Two-sided Mann-Whitney U-tests). Tukey boxplots are shown, where outer bounds of the box represent the first and third quartile, the centre line represents the median, and whiskers represents the nearest rounded whole number from 1.5 times the IQR below the first quartile and above the third quartile. A) Maxima and minima correspond with the whiskers. B) <45 maxima = 9.208, <45 minima = 0, >45 maxima = 9.876, <45 = 0. Source data are provided as a Source Data file.

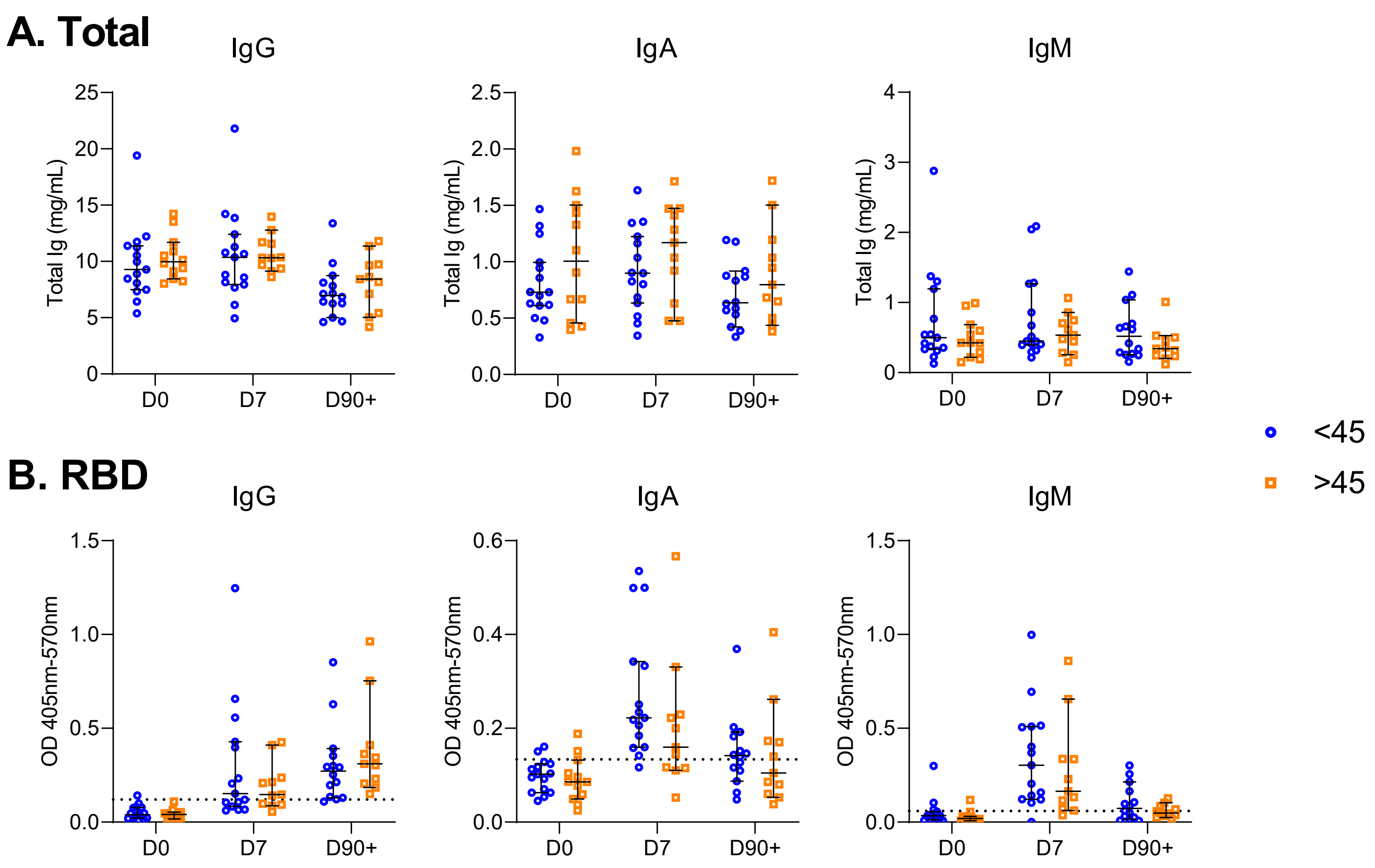

Supplementary Figure 6. Differences in A) total and B) RBD-specific antibody between patients below and above 45 years old at day 0, day 7, and day 90+. Dashed line in B) represents the mean + 2SD of results obtained from 8 pre-pandemic plasma controls collected in 2018-2019. Each dot represents a different patient. N values are as follows: <45, D0 (n = 15), <45, D7 (n = 15), <45, D90+ (n = 14), >45, D0 (n = 12), >45, D7 (n = 11), >45, D90+ (n = 11). No significant differences between groups were observed using two-sided Mann-Whitney U tests. Bar lines represent median and 95% CI. Source data are provided as a Source Data file.

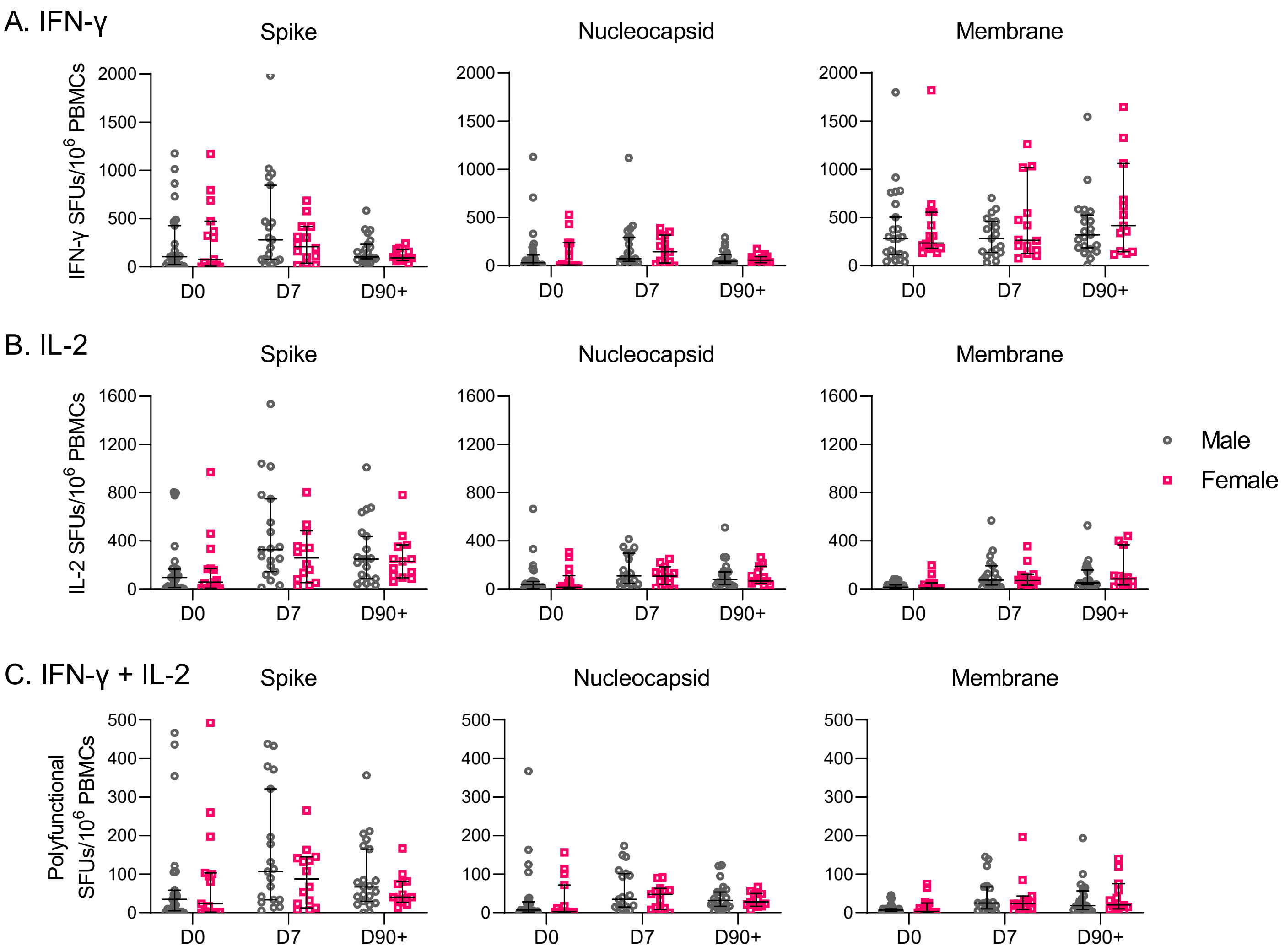

Supplementary Figure 7. Differences in T cell responses between sex at day 0, day 7, and day 90+. A) IFN-γ B) IL-2 C) Polyfunctional (IFN-γ + IL-2) T cell responses (as SFUs per 10^6 PBMCs) against structural SARS-CoV-2 protein peptide pools were compared between male and females. Each dot represents a different patient. N values are as follows: Male, D0 (n = 23), Male, D7 (n = 19), Male, D90+ (n = 21), Female, D0 (n = 15), Female, D7 (n = 14), Female, D90+ (n = 13). No significant differences between groups were observed using two-sided Mann-Whitney U tests. Bar lines represent median and 95% CI. Source data are provided as a Source Data file.

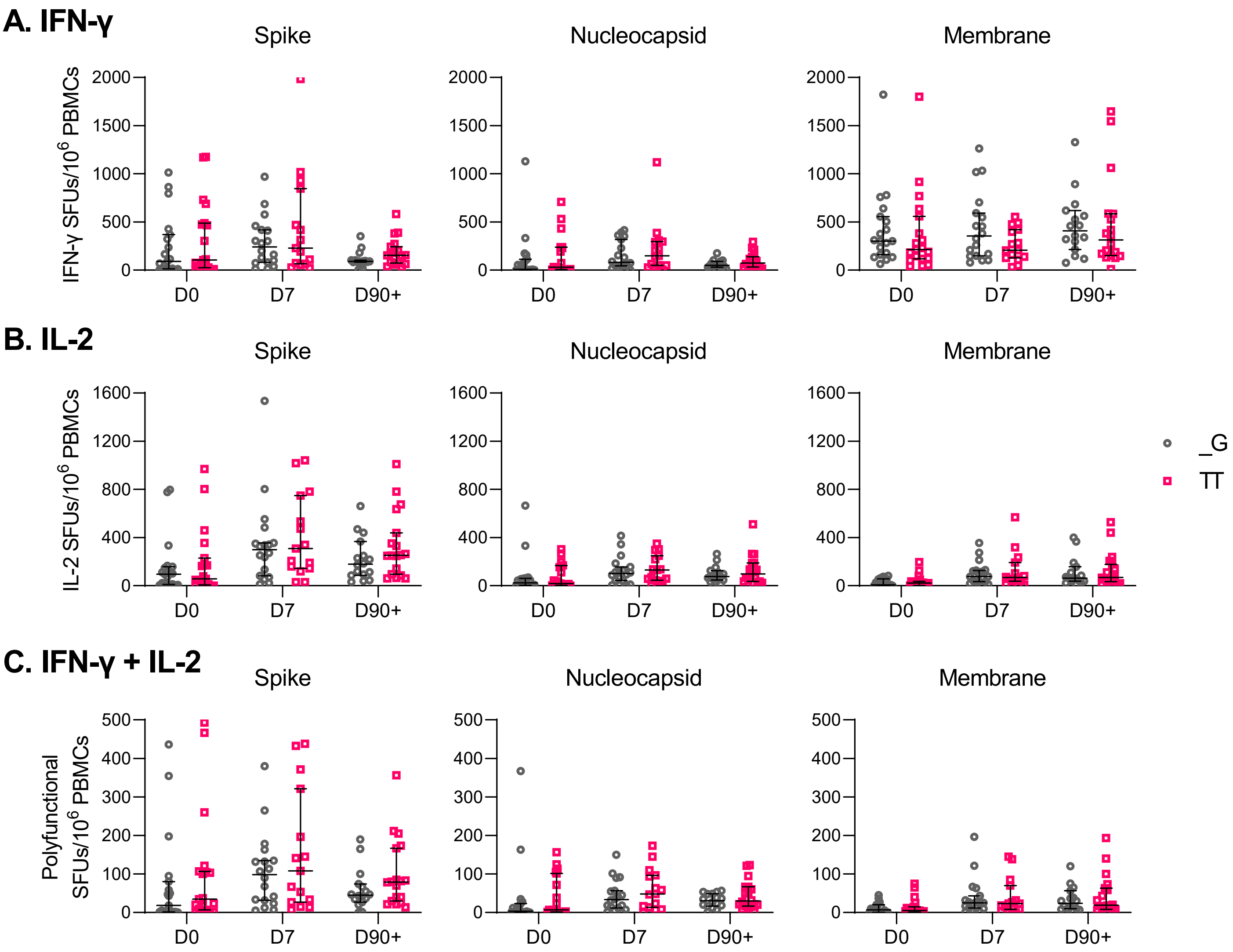

Supplementary Figure 8. Comparison of T cell responses between *IFNL4* genotype at day 0, day 7, and day 90+ post-enrollment. A) IFN-γ B) IL-2 C) Polyfunctional (IFN-γ + IL-2) T cell responses (as SFUs per 10^6 PBMCs) against structural SARS-CoV-2 protein peptide pools were compared between genotypes. "_G" indicates non-TT rs368234815 polymorphisms at the IFNL4 locus. Each dot represents a different patient. N values are as follows: _G, D0 (n = 19), _G, D7 (n = 18), _G, D90+ (n = 16), TT, D0 (n = 19), TT, D7 (n = 15), TT, D90+ (n = 18). No significant differences between groups were observed using two-sided Mann-Whitney U tests. Bar lines represent median and 95% CI. Source data are provided as a Source Data file.

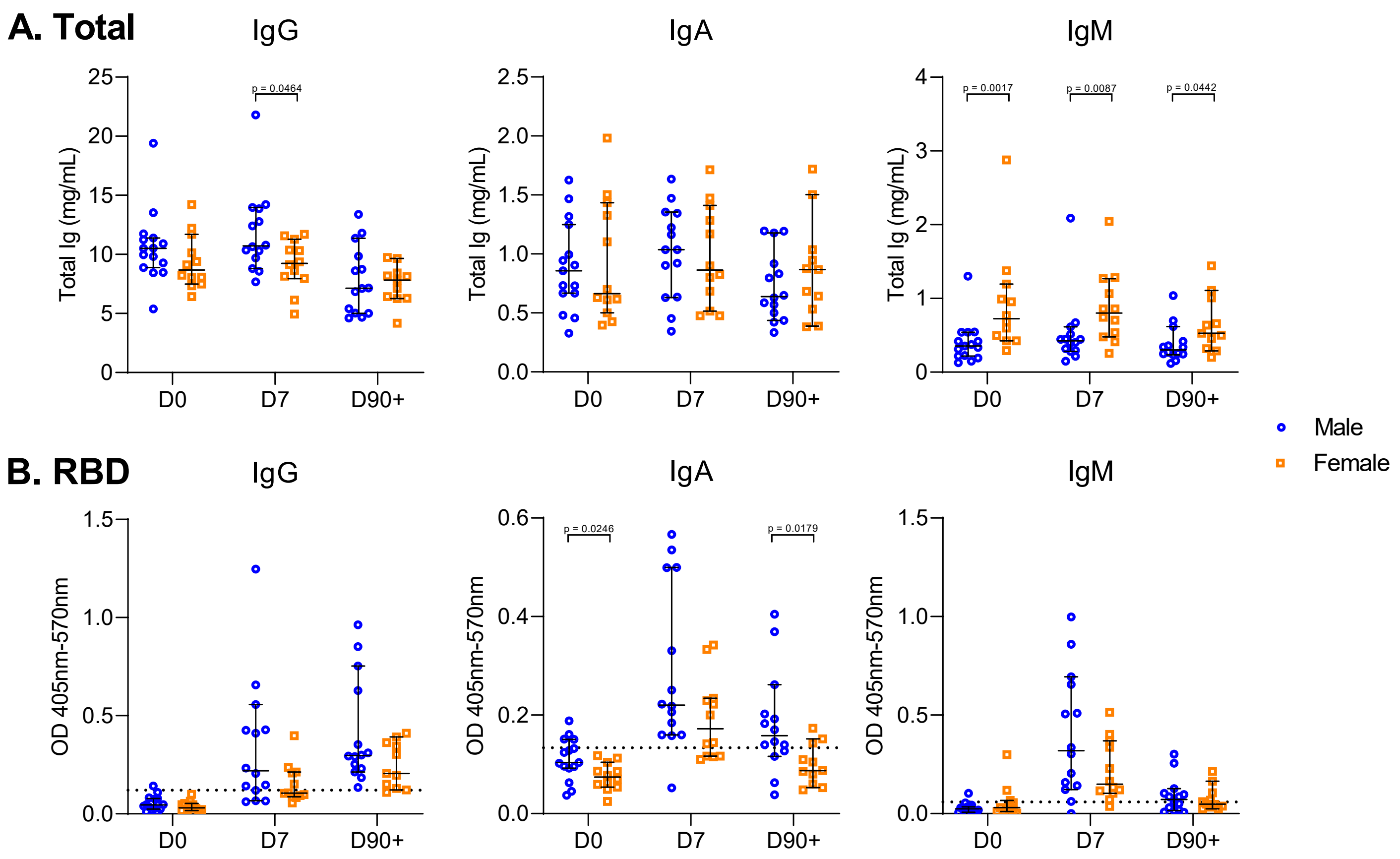

Supplementary Figure 9. Differences in A) total and B) RBD-specific antibody levels between sex at day 0, day 7, and day 90+. Dashed line in B) represents the mean + 2SD of results obtained from 8 pre-pandemic plasma controls collected in 2018-2019. Each dot represents a different patient. N values are as follows: Male, D0 (n = 15), Male, D7 (n = 14), Male, D90+ (n = 14), Female, D0 (n = 12), Female, D7 (n = 12), Female, D90+ (n = 11). Significant differences were observed using two-sided Mann-Whitney U-tests between sexes. Bar lines represent median and 95% CI. Source data are provided as a Source Data file.

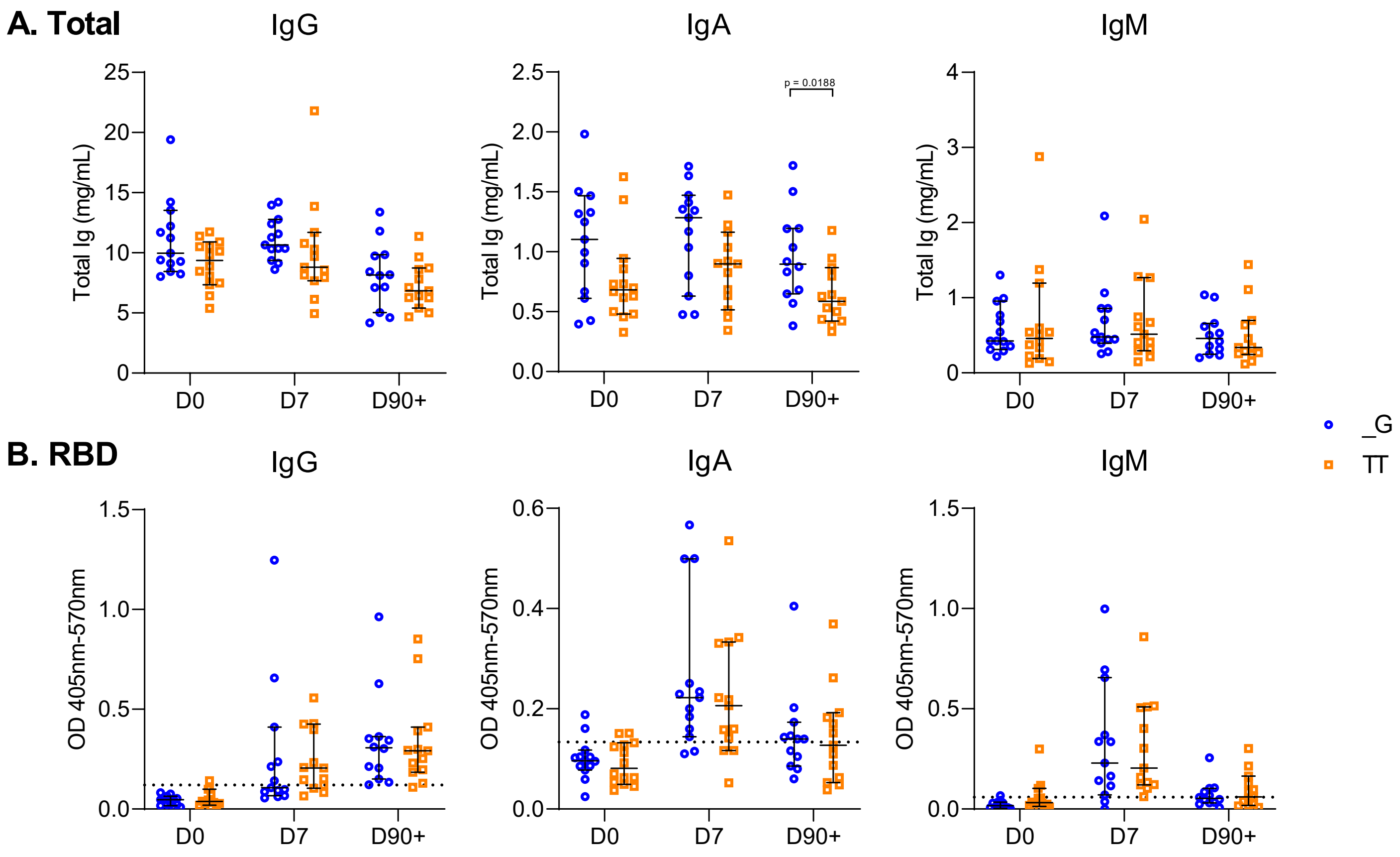

Supplementary Figure 10. Differences in A) total and B) RBD-specific antibody levels between *IFNL4* genotype at day 0, day 7, and day 90+. Significant differences were observed using Mann-Whitney U-tests between *IFNL4* genotypes (* $p < 0.05$). Each dot represents a different patient. N values are as follows: _G, D0 (n = 13), _G, D7 (n = 13), _G, D90+ (n = 12), TT, D0 (n = 14), TT, D7 (n = 13), TT, D90+ (n = 13). Significant differences were observed using two-sided Mann-Whitney U-tests between genotypes. Bar lines represent median and 95% CI. Source data are provided as a Source Data file.

Supplementary Table 5. SARS-CoV-2 structural protein peptide pools.

Protein	Peptide Pool Name	# of peptides	Pool Length
Membrane	M	31	1-222
Envelope	E	10	1-75
Nucleocapsid	N1	30	1-220
	N2	29	211-419
Spike	S1	45	1-325
	S2	45	316-640
	S3	45	631-955
	S4	46	946-1273
