# Supplementary material for: Interferon-λ treatment accelerates SARS-CoV-2 clearance despite age-related delays in the induction of T cell immunity
Source: Nat Commun. 2022 Nov 16;13:6992. doi: 10.1038/s41467-022-34709-4 (PMC9667439; doi:10.1038/s41467-022-34709-4)
Supplement: Supplementary file 3 — Reporting Summary [file 41467_2022_34709_MOESM3_ESM.pdf]

nature portfolio

Corresponding author(s): Adam Gehring, Deanna Santer

Last updated by author(s): Oct 17, 2022

Reporting Summary

Nature Portfolio wishes to improve the reproducibility of the work that we publish. This form provides structure for consistency and transparency in reporting. For further information on Nature Portfolio policies, see our Editorial Policies and the Editorial Policy Checklist.

Statistics

For all statistical analyses, confirm that the following items are present in the figure legend, table legend, main text, or Methods section.

n/a	Confirmed	
☐	☒	The exact sample size (*n*) for each experimental group/condition, given as a discrete number and unit of measurement
☐	☒	A statement on whether measurements were taken from distinct samples or whether the same sample was measured repeatedly
☐	☒	The statistical test(s) used AND whether they are one- or two-sided<br>*Only common tests should be described solely by name; describe more complex techniques in the Methods section.*
☒	☐	A description of all covariates tested
☐	☒	A description of any assumptions or corrections, such as tests of normality and adjustment for multiple comparisons
☐	☒	A full description of the statistical parameters including central tendency (e.g. means) or other basic estimates (e.g. regression coefficient) AND variation (e.g. standard deviation) or associated estimates of uncertainty (e.g. confidence intervals)
☐	☒	For null hypothesis testing, the test statistic (e.g. F, t, r) with confidence intervals, effect sizes, degrees of freedom and P value noted<br>*Give P values as exact values whenever suitable.*
☒	☐	For Bayesian analysis, information on the choice of priors and Markov chain Monte Carlo settings
☒	☐	For hierarchical and complex designs, identification of the appropriate level for tests and full reporting of outcomes
☐	☒	Estimates of effect sizes (e.g. Cohen's *d*, Pearson's *r*), indicating how they were calculated

Our web collection on statistics for biologists contains articles on many of the points above.

Software and code

Policy information about availability of computer code

Data collection	CTL ImmunoSpot Software 5.1
Data analysis	GraphPad Prism version 9.3, 10X Genomics Cell Ranger 6.0.0, R version 4.1.1 (Seurat v4.0.4, scDblFinder v1.7.464, ggplot2 v3.3.5, ggrepel v0.9.1, patchwork v1.1.1, dplyr v1.0.7, reshape2 v1.4.4, Harmony v0.1.0)

For manuscripts utilizing custom algorithms or software that are central to the research but not yet described in published literature, software must be made available to editors and reviewers. We strongly encourage code deposition in a community repository (e.g. GitHub). See the Nature Portfolio guidelines for submitting code & software for further information.

Data

Policy information about availability of data

All manuscripts must include a data availability statement. This statement should provide the following information, where applicable:

- Accession codes, unique identifiers, or web links for publicly available datasets
- A description of any restrictions on data availability
- For clinical datasets or third party data, please ensure that the statement adheres to our policy

The single-cell RNA-sequencing data generated in this study have been deposited in the Gene Expression Omnibus (GEO) database under accession code GSE215814. Source Data are provided with this paper.

Human research participants

Policy information about studies involving human research participants and Sex and Gender in Research.

Reporting on sex and gender	Sex as a biological variable was specifically analyzed in this study (Supplementary Figure 7, 9) and reported male/females in each group are shown in Table 1.
Population characteristics	We show age, sex and IFNL4 genotype in Table 1 for all participants studied.
Recruitment	Clinical trial recruitment is reported in previous Feld et al. Lancet Respiratory Medicine 2021 paper
Ethics oversight	University of Toronto, University of Alberta and University of Manitoba

Note that full information on the approval of the study protocol must also be provided in the manuscript.

Field-specific reporting

Please select the one below that is the best fit for your research. If you are not sure, read the appropriate sections before making your selection.

- [x] Life sciences
- [ ] Behavioural & social sciences
- [ ] Ecological, evolutionary & environmental sciences

For a reference copy of the document with all sections, see nature.com/documents/nr-reporting-summary-flat.pdf

Life sciences study design

All studies must disclose on these points even when the disclosure is negative.

Sample size	The sample size for the study was based on power calculations to show a virological effect of peginterferon lambda, with an expectation of 40% viral clearance by Day 7 in the placebo arm and 80% in the peginterferon-lambda arm, requiring 30 patients per arm, to achieve 80% power with alpha of 0.05, accounting for 10% dropout. The results were in line with the sample size estimates in the population with a high baseline viral load (79% clearance in peginterferon lambda arm vs 38% in the placebo arm) and showed a clear acceleration of viral clearance with treatment compared to placebo. For this ancillary study of immune responses, we used as many samples remaining as possible from consenting participants. The scRNAseq analysis was limited to 9 study participants due to sample availability and cost (3 time points each). Notably, although this scRNAseq sample size is small, it is in line with many other studies using single cell analyses.
Data exclusions	No data was excluded.
Replication	For all fluorospot and ELISA analyses, replicates of all samples were performed and results were consistent across technical and biological replicates.
Randomization	For the clinical trial our samples were collected from: Eligible consenting adults were randomized 1:1 to a single subcutaneous injection of 180µg of peginterferon-lambda or saline placebo. A computer-generated randomization list was created by the study statistician with a randomization schedule in blocks of four. At the time of randomization, the study personnel received a sealed opaque envelope with the treatment allocation number that indicated which vial to administer to the participant. Study medications were stored in individual, numbered opaque bags in the study refrigerator until use. Analysis of viral clearance was controlled for baseline viral load given the variability and the impact on time to clearance. Randomization was successful for other relevant covariates with well-matched groups.
Blinding	Investigators were not blinded during data analysis, but plasma samples were added to ELISAs and PBMCs were added to fluorospot plates blinded.

Reporting for specific materials, systems and methods

We require information from authors about some types of materials, experimental systems and methods used in many studies. Here, indicate whether each material, system or method listed is relevant to your study. If you are not sure if a list item applies to your research, read the appropriate section before selecting a response.

Materials & experimental systems

n/a	Involved in the study
☐	☒ Antibodies
☒	☐ Eukaryotic cell lines
☒	☐ Palaeontology and archaeology
☒	☐ Animals and other organisms
☒	☐ Clinical data
☒	☐ Dual use research of concern

Methods

n/a	Involved in the study
☒	☐ ChIP-seq
☒	☐ Flow cytometry
☒	☐ MRI-based neuroimaging

Antibodies

Antibodies used

1. Donkey anti-human IgG Fcgamma specific (Jackson Immuno #709-005-098)
2. ChromPure Human IgG (Jackson Immuno #009-000-003)
3. Goat anti-human IgG (Fcg)-Alkaline phosphatase conjugated (Jackson Immuno #109-055-190)
4. Donkey anti-human IgM (Jackson Immuno #709-005-073)
5. ChromPure Human IgM (Jackson Immuno #009-000-012)
6. Goat anti-human IgM (Fc5u)-Alkaline phosphatase conjugated (Jackson Immuno #109-055-129)
7. Goat anti-human IgA (α chain)- Alkaline phosphatase conjugated (Invitrogen #A18784)
8. Human IgA Antibody Pair kit-(Stemcell Technologies #01992)

Validation

Validation of all antibodies was performed by the respective manufacturers they were purchased from.

Jackson Immunoresearch:
1 and 3. Based on immunoelectrophoresis and/or ELISA, the antibody reacts with the Fc portion of human IgG heavy chain but not with the Fab portion of human IgG. No antibody was detected against human IgM or IgA, or against non-immunoglobulin serum proteins.
2. Purity: Based on immunoelectrophoresis at an antigen concentration of 20 mg/ml, the pattern of precipitation against goat anti-human whole serum is the same as that against goat anti-human IgG, Fc fragment specific. No precipitin line was detected against goat anti-human IgM, Fc5µ fragment specific or goat anti-human IgA, a chain specific.
4 and 6. Based on immunoelectrophoresis and/or ELISA, the antibody reacts with the Fc5µ portion of the human IgM heavy chain but not with human IgG, IgA, or the light chains of human immunoglobulins. No antibody was detected against non-immunoglobulin serum proteins.
5. Purity: Based on immunoelectrophoresis at an antigen concentration of 20 mg/ml, the pattern of precipitation against goat anti-human whole serum is the same as that against goat anti-human IgM, Fc5µ fragment specific. No precipitin line was detected against goat anti-human IgG, Fc fragment specific; goat anti-human IgA, a chain specific; or goat anti-humanalpha-2 macroglobulin.

Invitrogen:
7. The sensitivity of each lot of antibody is confirmed using ELISA. The specificity of each lot of antibody is confirmed by immunoelectrophoresis (IEP).

STEMCELL Technologies https://www.stemcell.com/products/product-types/elisa-kits-overview.html
8. Highly specific antibodies detect analytes in the low pg/mL range and standards are calibrated against international references.
